# Artificial Intelligence in Psychiatry: A New Paradigm

**DOI:** 10.1192/j.eurpsy.2024.442

**Published:** 2024-08-27

**Authors:** M. Gerantia

**Affiliations:** Center of mental Health and addiction prevention, Tbilisi, Georgia

## Abstract

**Introduction:**

The advent of artificial intelligence (AI) and machine learning has sparked interest in its applicability in the mental health domain, offering potential improvements in the efficiency and personalization of psychiatric services.

**Objectives:**

To characterize the methodological and technical approaches in studies utilizing machine learning and natural language processing (NLP) within mental health, to evaluate their potential and impact in psychiatric clinical practice, and to address the associated ethical concerns.

**Methods:**

A systematic review, adhering to the PRISMA guidelines, was conducted across four primary medical databases. Emphasis was placed on studies that applied machine learning and NLP techniques to psychiatric contexts, extracting data from sources such as medical records and social media.

**Results:**

From 327 identified articles, 58 were considered relevant. Major themes included symptom extraction, illness severity classification, therapy effectiveness comparison, and psychopathological insight derivation. Notably, most studies focused on specific populations like social media users, emergency room attendees, or those within medical databases. Methodological findings showcased a preference for efficient classifiers and Python as the primary platform.

**Conclusions:**

Machine learning and NLP offer a promising new avenue for psychiatric research and clinical practice, enabling the extraction of previously inaccessible patient information and supporting the decision-making process. However, the field must address inherent limitations, ethical considerations, and ensure that the tools augment, rather than replace, clinical judgment.

**Disclosure of Interest:**

None Declared

